# ^1^H-NMR Profiling Shows as Specific Constituents Strongly Affect the International EVOO Blends Characteristics: The Case of the Italian Oil

**DOI:** 10.3390/molecules26082233

**Published:** 2021-04-13

**Authors:** Francesca Calò, Chiara Roberta Girelli, Federica Angilè, Laura Del Coco, Lucia Mazzi, Daniele Barbini, Francesco Paolo Fanizzi

**Affiliations:** 1 Department of Biological and Environmental Sciences and Technologies, University of Salento, Prov.le Lecce-Monteroni, 73100 Lecce, Italy; francesca.calo@unisalento.it (F.C.); chiara.girelli@unisalento.it (C.R.G.); federica.angile@unisalento.it (F.A.); laura.delcoco@unisalento.it (L.D.C.); 2 Certified Origins Italia S.r.l., Località il Madonnino, 58100 Grosseto, Italy; lucia.mazzi@oleificioolma.it (L.M.); daniele.barbini@certifiedorigins.it (D.B.)

**Keywords:** ^1^H-NMR spectroscopy, extra virgin olive oil, multivariate statistical analysis, traceability

## Abstract

Considering the growing number of extra virgin olive oil (EVOO) producers in the world, knowing the influence of olive oils with different geographical origins on the characteristics of the final blend becomes an interesting goal. The present work is focused on commercial organic EVOO blends obtained by mixing multiple oils from different geographical origins. These blends have been studied by ^1^H-NMR spectroscopy supported by multivariate statistical analysis. Specific characteristics of commercial organic EVOO blends originated by mixing oils from Italy, Tunisia, Portugal, Spain, and Greece were found to be associated with the increasing content of the Italian component. A linear progression of the metabolic profile defined characteristics for the analysed samples—up to a plateau level—was found in relation to the content of the main constituent of the Italian oil, the monocultivar Coratina. The Italian constituent percentage appears to be correlated with the fatty acids (oleic) and the polyphenols (tyrosol, hydroxytyrosol, and derivatives) content as major and minor components respectively. These results, which highlight important economic aspects, also show the utility of ^1^H-NMR associated with chemometric analysis as a powerful tool in this field. Mixing oils of different national origins, to obtain blends with specific characteristics, could be profitably controlled by this methodology.

## 1. Introduction

Extra virgin olive oil (EVOO) is the well-known product obtained from the fruit Olea europaea by mechanical cold pressing of olives. To date, EVOO remains undoubtedly the most important production of Mediterranean countries, due to its nutraceutical, antioxidant and other health properties [1,2,3,4]. Mediterranean countries are responsible for 98% of globally produced olive oil, including 105 registered within an EU quality scheme in EU countries (Croatia, France, Greece, Italy, Portugal, Slovenia, and Spain). According to the registered PDO/PGI (Protected Designation of Origin/Protected Geographical Indication) [5], Italy continues to hold the European quality record in EVOOs, but the commercial globalization of this product includes also countries outside the Mediterranean area [6]. Olive oil represents a significant percentage of the overall world fat consumption. The global demand for EVOO steadily grows due to its many beneficial properties, leading to new countries entering the global market as producers [3,7,8]. Properties can vary greatly in oils from different countries [9]. The quality of olive oil is the result of various factors including the pedoclimatic conditions that characterize the production place. For this reason, the geographical origin declaration, on the information label of this product, is commercially important [10]. Recently, there has been growing consumer interest in the EVOOs labelling which should report the declared varietal and geographical origin of the product [6]. Being a premium price product in the national and international market, EVOO’s authenticity and traceability are important for both consumer health and commercial purposes. Currently, the geographical origin and composition assessment of EVOOs blend remains mostly a self-regulatory code and granting procedures are currently based essentially on producer companies’ declarations. Proposals for new regulations are always under discussion to ensure the clarity required by official standardized procedures. In this context, it is necessary to guarantee food safety for high-value EVOOs through a scientific control method against frequent adulterations. In fact, EVOO is one of the most frequently counterfeit foods and this is often related to incorrect or misleading geographical origin declaration [8,11]. It has been suggested that, in order to develop reliable classification methods, the analytical techniques proposed for geographical origin assessment must refer to large databases containing characterizing data for a huge number of selected oils [12]. Clearly, this is quite complex, considering blends of multiple oils with different national origins. In recent years, this topic has attracted many scholars. Accordingly, many studies have been devoted to single country EVOOs [13], products contaminated by non-olive oils [14] as well as varietal origin assessment [15]. On the other hand, multi-country blends have been rarely explored, although they constitute a considerable market share. Taking into consideration the multiplicity of originating countries, whose oils are used to constitute blends, geographical and varietal traceability of the most representative olive oils in the world, used for blend production, is highly advisable, although very complex. Thus, the present study is focused on commercial organic EVOO blends obtained by mixing multiple oils from different countries. The oils constituting the considered blends have been characterized singularly in terms of fatty acids and polyphenols composition as reported in literature [16]. In fact, there are many studies related to EVOOs from Italy [1,17], Tunisia [18], Portugal [19], Greece [17,20], and Spain [21]. In particular, the aim of this work is to verify, following our studies on Italian EVOOs [7], the possibility of correlating a specific blend composition and characteristics with the geographical origin of the constituents, by using ^1^H-NMR metabolic profiles databases and MVA (multivariate analysis). This also in order to support commercial statements reported in the EVOOs labels. The NMR and MVA techniques have been extensively exploited for building statistical models allowing the differentiation of national EVOOs and, in particular, Italian EVOOs with respect to other products [22]. The obtained spectra allow distinguishing EVOOs from different European and non-European countries [10]. The NMR methodology has significant potential in controlling the authenticity and quality of olive oil, giving information complementary to classical analyses [23]. The highly reproducible and detailed ^1^H-NMR fingerprint also suggests the possible evaluation of different oils contribution inside a specific blend. Examples of this approach related to a laboratory produced binary mixture of Tunisian/Italian EVOOs blend have been already reported by Girelli et al. [8]. On the other hand, the purpose of this work is to investigate the possible correlation of the final product characteristics with the composition of a commercial multi-country blend originated by mixing oils from Italy, Tunisia, Portugal, Spain, and Greece. Therefore, in this preliminary work, only the producer defined compositions in the available commercial blends have been considered.

## 2. Materials and Methods

Chemicals: All chemical reagents for analysis were of analytical grade. CDCl3 (99.8 atom %D) and tetramethylsilane, TMS (0.03 *v*/*v*%) were purchased from Armar Chemicals (Döttingen, Switzerland).

### 2.1. Sample Preparation

The 77 commercial samples of organic EU and outside EU produced EVOO blends supplied by Certified Origins Italia S.r.l. were stored in sealed dark glass bottles at room temperature in the dark before analysis. These blends originated from the 2017/2018 to 2020/2021 olive harvesting years and were analysed as received within the production year. Each sample has different combinations of the percentages of oils with different national origin: Italian, Spanish, Greek, Portuguese, and Tunisian. Commercial oil samples were provided by the producers before bottling. The composition was declared in the shipping documents by the supplying company. A detailed description, from the lower to the higher percentage of these oils, is summarized in Table 1. Moreover, a total of 74 monocultivar Coratina and 41 non-Italian EVOOs samples were also supplied by Certified Origins Italia S.r.l. The Coratina oil was the characteristics monocultivar constituent of Italian EVOOs used in the international blends as declared by the supplier. The 41 non-Italian reference oils used as monocultivars from specific geographical origins were Tunisia, Portugal, Spain and Greece EVOOs based on Chemlali e Chetoui (Tunisian), Arbequina (Portuguese and Spanish) and Koroneiki (Greek). All the studied international EVOOs blends were commercial samples obtained by cold extraction according to the provider declaration. The oil samples were prepared for ^1^H-NMR analysis by taking about 140 μL microliters of oil and diluting them in deuterated chloroform (CDCl_3_), in order to respect the ratio of 13.5% oil 86.5% CDCl_3_, *w*/*w* [8]. Then, 600 μL of obtained mixture was inserted into a 5 mm diameter NMR tube about 20 cm long and used for spectroscopic analysis. The chemical shifts were expressed with respect to the resonance of the internal standard (TMS) contained in CDCl_3_ solvent.

### 2.2. ^1^H-NMR Analysis

The ^1^H-NMR analysis of the oil samples was carried out using a Bruker Avance III 400 MHz Nuclear Magnetic Resonance (NMR) spectrometer, equipped with a direct (BBO) and inverse (BBI) probe, with a 60-position autosampler. The acquisition of the NMR spectra took place in automation after inserting the samples into the BACS-60 autosampler (Bruker) interfaced with the IconNMR software (Bruker). For each sample, a 1D ^1^H-NMR analysis was performed to investigate the majority component (glyceridic component and fatty acids) with the following parameters: zg pulse program, 64k time domain, spectral amplitude 20.5555 (8223.685 Hz), p1 12.63 μs, pl1 − 1.00 db, 16 repetitions, 3.98 s acquisition times, 4 s relaxation delay between scans. In addition, the samples were examined with a multi-suppressed (suppression of strong fatty acids signals) experiment to highlight also the minority component (polyphenols, aldehydes, terpenes, sterols), following the parameters: noesygpps1d.comp2 pulse program, 32k time domain, spectral amplitude 20.5555 (8223.685 Hz), p1 12.63 μs, pl1 − 1.00 db, 32 reps, 1.99 s acquisition times, 4 s relaxation delay between scans. The standard Bruker routine (ATMA, LOCK and TOPSHIM) was used to optimize the work conditions. The chemical shifts of the sample signals were calculated with respect to that of the internal standard (TMS), the signal of which was set at 0 ppm [24]. The internal standard as well as Glycerol [25] signals were also used for integral calibrations. The metabolites were identified based on 1D NMR spectra analysis and comparison with published data [26,27,28]. The NMR spectra were acquired and processed using Topspin 2.1 (Bruker) software. Then, each NMR spectrum was bucket reduced (0.04 ppm width) and further processed (Pareto scaled) using Amix software vers. 3.9.13 (Bruker Biospin). Bucketing of ^1^H zg NMR spectra was performed within the range 10.0–0.5 ppm and ^1^H noesygpps NMR spectra was carried out within the range 10.0–5.6 ppm. The region between 7.6 and 6.9 ppm was excluded to eliminate the area of the peaks of the residual solvent signal from the analysis. In order to minimize small differences due to olive oil concentration and/or experimental conditions among samples, the total sum normalization was then applied [29].

### 2.3. Multivariate Statistical Analysis Applied to NMR Data

The buckets reduced NMR spectra were used as input data for multivariate statistical analysis (MVA) by using the Simca-P version 14 software (Umetrics). The use of chemometric methods allows to detect and verify the “clustering” of the samples according to specific characteristics such as the EVOOs country of origin. Exploratory PCA (considering individual samples), PCA based partial least squares regression (PLSR) and a class discriminating analysis (OPLS-DA) (considering sample classes) were used in the present study. Unsupervised PCA (principal component analysis) is at the basis of the multivariate statistical analysis [30]. It is usually applied to extract and show the systematic variation in a data matrix X formed by rows (the considered observations, in our case olive oils NMR spectra) and columns (the variables which describe the samples, in our case the buckets from each NMR spectra). PCA models are developed to reduce the dimensionality of the data to obtain a series of variables that are optimized and easy to interpret [31]. Linear combinations of the original data variables are called main components (PCs). By PCA, groups of observations, trends and outliers that could be excluded from further analysis may be recognized. In addition, supervised PLS-DA (Partial least squares discriminant analysis) [32] or OPLS-DA (orthogonal partial least squares-discriminant analysis; used in the present study) [33] models are generally created for the discrimination between samples grouped in classes with different characteristics. PLS-DA is performed by rotating the main components (the axes that express the variance of the data), in order to obtain the maximum separation between groups of observations and information about the variables responsible for the observed separation [34]. OPLS-DA is a modification of the PLS-DA technique which filters out variation not directly related to the discriminating response. In details, OPLS-DA is realized by separating the portion of the variance useful for predictive purposes from the non-predictive variance (which is made orthogonal). The result is a model characterized by an improved interpretability [35]. The efficiency of the models is described by parameters R^2^ (cum) and Q^2^ (cum) related to the used minimum number of components (cum). R^2^ is used to have a quantitative measure of the described data variability and Q^2^ indicates the goodness of a prediction [36]. Considering the trend of the blends observed in the PCA, partial least squares regression (PLSR) models were also elaborated to possibly gain a quantitative classification, parallel to qualitative investigation. These models have obtained through the PLS (projection to latent structures by partial least squares) regression technique (Simca-P tool). This relates the X matrix of the dependent variables (in the present case the increasing percentage for a constituent from a selected country in the final blend) with the Y matrix of the independent variables (predicted percentage based on buckets reduced NMR spectra analysis) [8,37]. The predictivity measure for the model, called root mean square error from cross-validation (RMSECV), is found summarizing the cross-validation residuals of the observations in the workset. RMSECV can be regarded as an intermediary to root mean square error of estimation (RMSEE) and root mean square error of prediction (RMSEP). The internal cross-validation default method (7-fold) and the permutation test (40 permutations), both available on the SIMCA-P software [36,38], were used in order to validate the statistical models. Overall, this work shows PCA and OPLS-DA models with the relative characteristics that allow the characterization and typing of organic EU and outside EU produced EVOO blends showing their progress based on the constituent oils.

## 3. Results and Discussion

### 3.1. Unsupervised Analysis

**Figure 1:**
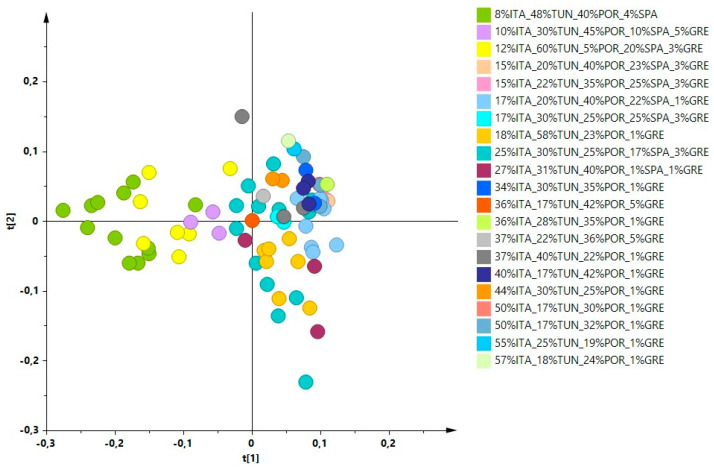
Score plot of the PCA model of ^1^H-NMR standard experiment data for samples of considered organic EU community and non-community oils. Colour coding indicates the specific blends; 3 components model with R^2^X = 0.854, Q^2^ = 0.773.

**Figure 2:**
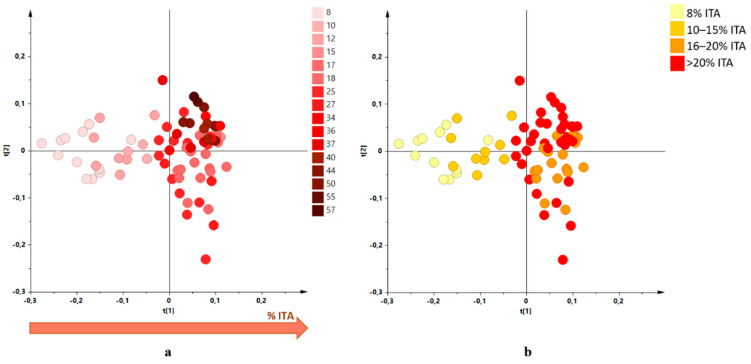
(**a**) Score plot of the Figure 1 PCA model showing the samples colour coding according to the increased percentages of Italian oil. (**b**) Score plot of the Figure 1 PCA model simplified to four classes colour coding: 8%, 10–15%, 16–20%, >20%.

Investigations, by ^1^H-NMR metabolic profiling, were carried out on organic EU and outside EU produced EVOOs, in particular to obtain information regarding the influence of the constituent oils percentages on the characteristics of the blend. The EVOOs samples were received from the supplier over four different harvesting years (from 2017/2018 to 2020/2021) and analysed as received. These blend samples, containing EVOOs from different countries in different percentage (Table 1), were analysed by ^1^H-NMR spectroscopy. All the statistical models were elaborated using the buckets reduced NMR spectra obtained for the blend samples. An unsupervised analysis (PCA) was performed using the NMR data of the analysed blends giving a well-defined score plot (Figure 1) for a three components model (R^2^X (cum) = 0.854 and Q^2^ (cum) = 0.773). Dealing with commercial EVOOs, the different percentages of the analysed samples were defined and declared by the international blend producers. Moreover, the number of samples for each specific international blend varied from 1 to 13 and was essentially correlated to the sampling frequency used by the bottling company according to specific blend use. After analysing in detail the PCA score plot obtained for all the commercial international blends, we realized that a specific blend characteristic (increasing percentage of Italian constituent) appeared to be correlated with the first component (PC1) in the unsupervised MVA. In order to simplify the Figure 1 score plot reading, the analysed blends were represented according to the increasing Italian oil content. A specific colour code showing the observed trend (deeper the colour higher the Italian oil percentage) was used to indicates the samples. As a result, the possible correlation of the Italian oil content with the PC1 values could be suggested (Figure 2a). A similar trend was not observed when all the other countries (Tunisia, Portugal, Spain, Greece) components were highlighted with the same approach (Figure S1). A similar investigation was also used considering pairwise joint components percentage analysis. In this case progression along the PC1 was only observed considering Italian and Greek components joint contribution (possibly for the very limited Greek oil presence 1–5%). No clear correlation with PC1 for the pairwise joint components percentage was observed for all the other cases (Supplementary Figure S1). A detailed analysis was performed considering the increase of the dominant Italian components for all available increasing percentages ranges. For clarity, in the PCA score plot analysis, a limited number of percentage ranges (8%, 10–15%, 16–20%, >20% ITA) was further used as colour coding giving even better results (Figure 2b). Indeed, the final blend characteristics dependence upon the ITA percentage seems to undergo plateauing over a 20% value.

### 3.2. Linear Regression Analysis

**Figure 3:**
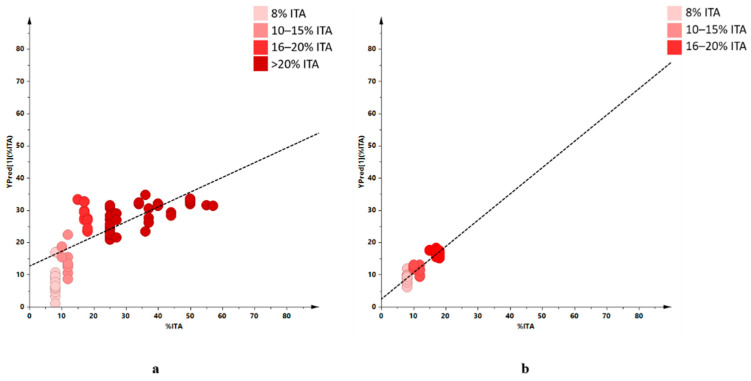
(**a**) The partial least squares regression (PLSR) model for organic European community and non-community extra virgin olive oil (EVOO) blend samples as a function of the percentage of Italian oil calculated for each blend sample. One component, R^2^X (cum) = 0.662, R^2^Y (cum) = 0.816 and Q^2^ (cum) = 0.809, goodness of fit R^2^ = 0.816. Root mean square error of cross-validation (RMSECV)(Y) = 1.7766. (**b**) The PLSR in Figure 3a limited to the specific range 8–20% ITA.

Partial least squares regression (PLSR) analysis, parallel to the qualitative investigation of the PCA models, was also performed to possibly gain a quantitative classification trend. The increasing percentage for a constituent from a selected country, in the final blend, was therefore correlated with the Y matrix of the independent variables (predicted percentage based on buckets reduced NMR spectra analysis). In detail, the percentages of the constituents from a selected country were placed as the experimental variables on the abscissa axis and the predicted values were reported as Y Pred on the ordinate axis (Supplementary Figure S2). The observed trend clearly showed a linear dependence in the range 8–20% ITA and a plateauing effect for higher ITA compositions (Figure 3a). By limiting the PLSR analysis to the specific range 8–20% ITA (Figure 3b) a linear relationship between the two matrices X and Y resulted as expressed by the linear function y = 0.816x + 2.405 with a correlation coefficient R^2^ = 0.816. The parameters of the PLSR model with one component are R^2^X (cum) = 0.662, R^2^Y (cum) = 0.816 and Q^2^ (cum) = 0.809, respectively for descriptive and predictive capacity of the system. The overall result could be considered satisfactory since the variable errors Y (RMSECV) (Y) = 1.7766 also represent the heterogeneous composition of the blend consisting of oils from different nationalities, in addition to the Italian. Thus, Italian oil in the range of 8–20% (before the plateau level) shows good linear dependence of the ^1^H-NMR profiling predicted with the actual percentages (R^2^ = 0.816). Interestingly, this specific best performing correlation is observed only when considering the increasing ITA constituent percentage in the blends. Moreover, the described best linear dependence by increasing ITA only percentage also holds, with respect to similar correlations observed for all the other countries, when the plateau is included (Supplementary Figure S2). Analysis of the R^2^ values in the corresponding regression lines accounts for this result (Table 2).

### 3.3. Metabolic Profile

**Figure 4:**
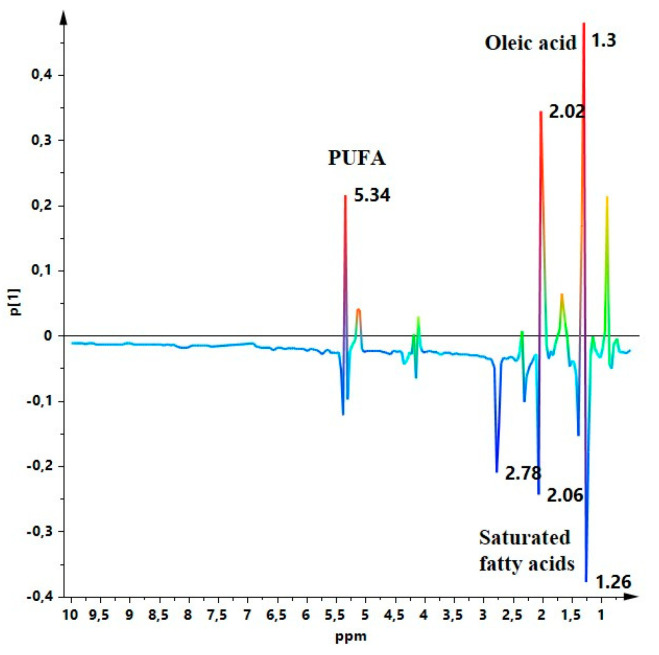
Line-plot of the Figure 1 PCA model for samples of considered organic EU community and non-community oils; 3 components model with R^2^X = 0.854, Q^2^ = 0.773.

**Figure 5:**
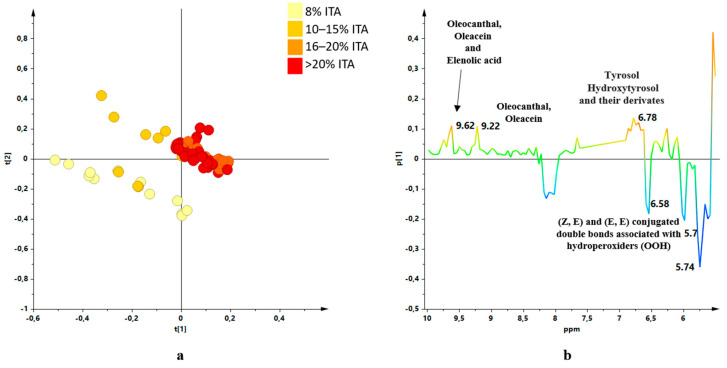
(**a**) Score plot of the PCA model of ^1^H-NMR multi-suppressed experiment data for samples of considered organic EU community and non-community oils; 3 components model with R^2^X = 0.730, Q^2^ = 0.610. (**b**) S-line plot for the Figure 5a PCA model.

**Figure 6:**
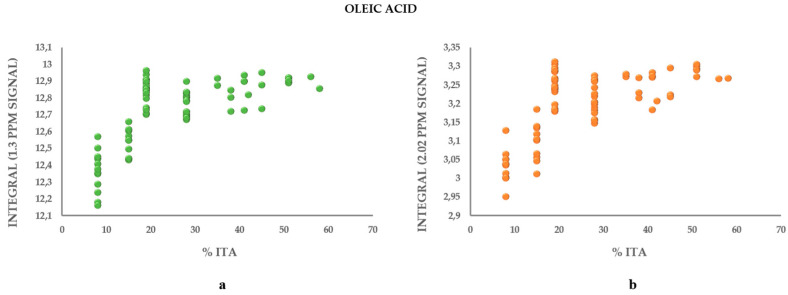
Integral of the area underlying the peaks corresponding to oleic acid in the NMR spectrum (**a**) 1.03 and (**b**) 2.02 ppm correlated with the Italian component in the blends.

**Figure 7:**
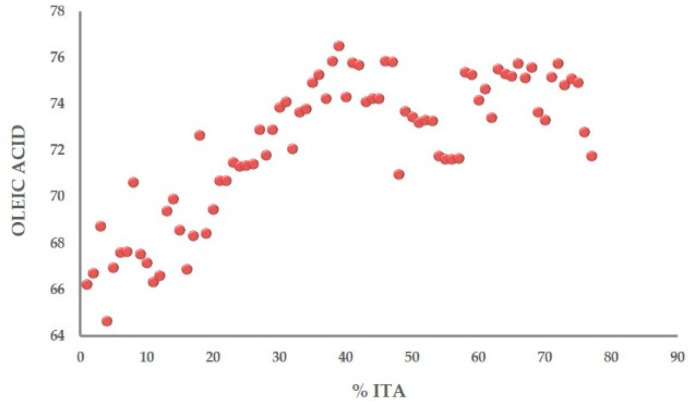
Quantification of oleic acid content correlated with the Italian component in the blends.

**Figure 8:**
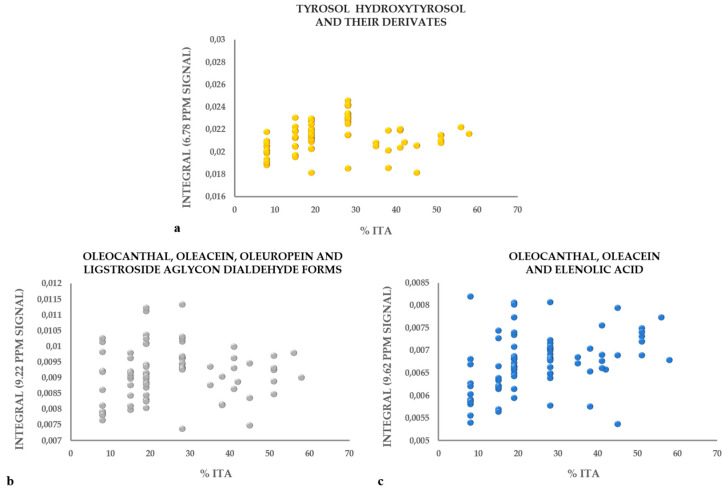
Integral of the area underlying the peaks corresponding to polyphenols in the NMR spectrum—tyrosol, hydroxytyrosol and their derivatives (**a**) 6.78 ppm, oleocanthal, oleacein, oleuropein and ligstroside aglycon dialdehyde forms (**b**) 9.22 ppm and, oleocanthal, oleacein and elenolic acid (**c**) 9.62 ppm—correlated with the Italian component in the blends.

In order to identify the samples metabolites responsible for the observed variation in the first component of the obtained PCA model, the corresponding Loading Line plot was analysed (Figure 4). Considering the EVOOs major components, an increase in the content of oleic acid (1.03, 2.02 ppm) and unsaturated fatty acids (5.34 ppm) appears correlated to higher percentages of Italian oil. On the contrary, increased saturated fatty acids (1.26 ppm) [27] appears to be correlated to a lower share of Italian oil. In order to enhance minor components contribution to the studied organic EVOOs blend, a multi-suppressed ^1^H-NMR (with suppression of strong fatty acid signals) experiments were performed. Also in this case, the obtained PCA model showed a distribution of international EVOO blend samples with a slight progression on the PC1 according to the Italian oil content (although limited to the first part of the focused component; Figure 5a). The PC1 S-line plot for the model indicated the molecular components responsible for the differentiation along the first component as essentially related to aromatic groups (Figure 5b). Indeed, the EVOOs polyphenolic component slightly increases as the content of Italian oil increases in the blends essentially in the range 8–20% reaching thereafter a nearly constant concentration. In detail, a modest rise in tyrosol, hydroxytyrosol and their derivatives (6.78 ppm) was observed together with aldehyde residues ascribable to oleocanthal (ρ-HPEA-EDA; 9.22 and 9.62 ppm) as well as oleacein, oleuropein and ligstroside aglycon dialdehyde forms [26] and, possible presence of elenolic acid (9.62 ppm) normally found in much lower concentration than oleacein or oleocanthal [26,39,40,41,42,43]. The chemical shifts of the minor molecular component, considered in this study, are relative of the groups of polyphenolic compounds highlighted by the S-line plot and identified by spectra analysis [44] for the studied international EVOOs blends. Tyrosol, hydroxytyrosol, and their derivatives belong to the Secoiridoids (SEC) phenolic compounds. Oleocanthal and ligstroside aglycon are the esterified derivates of tyrosol, oleacein and oleuropein are the esterified derivates of hydroxytyrosol [26,44] and elenolic acid is a product derived from oleuropein [45] or ligstroside aglycon monoaldehydic form [46] hydrolysis. As stated before, the overall content in polyphenols remains roughly constant for percentages of Italian oil in the blends >20%. Interestingly, in accord with lack of antioxidant characteristics of the aromatic polyphenol component, there is an increase content in (Z, E) and (E, E) conjugated double bonds associated with hydroperoxides (OOH) (6.58, 5.7, 5.74 ppm) in the international blend samples with a lower content of Italian oil. The hydroperoxides show signals indicating a certain oxidation already undergone for EVOOs samples (possibly during processing and/or storage) [26]. Representative ^1^H-NMR spectra and chemical shift data of major and minor components are reported in Supplementary Figure S3. According to these observations, the blend characteristics conferred by the Italian oil are due to the major and minor EVOO molecular components. In particular, the major components (essentially oleic acid) seem to prevail. As shown in Figure 6, the integral of the area underlying the peaks corresponding to oleic acid in the NMR spectrum show a clear correlation with the Italian component in the blends very similar to that observed in Figure 3 where the EVOOs complete profiles is considered. Fatty acids composition for the examined commercial international blends, calculated by NMR data, according to the published procedures [25], is reported in Supplementary Table S1. The obtained composition data resulted in accord with ranges reported for other EVOOs in literature [25,47,48]. In particular, the oleic acid content shows a clear correlation between the obtained values and the Italian component in the blends. This specific correspondence is also in accord with the linear trend in the range 8–20% ITA and the plateau for higher ITA compositions (Figure 7). On the other hand, also the minor molecular components certainly play a role by giving characteristics related to the presence of polyphenols to the blends (Figure 8). Moreover, the difference of the integral values observed at 9.22 ppm and 9.62 ppm is an indication of more nearly-overlapping signals occurring in the former case due to the presence of oleuropein and ligstroside aglycon dialdehyde forms which are major ingredients in Coratina variety [49] rather than a major presence of elenolic acid at 9.62 ppm [26] (Figure 8). These major and minor molecular components contributions to the blend characteristics seems to plateau out for due to the Italian oil concentrations greater than 20%. This may have a meaning, since Italian oils with lower oleic and polyphenol contents could be used in larger quantities than oils with higher polyphenol contents, resulting in similar final characteristics of the blends. Indeed, an optimal level of bitterness is usually achieved finely trimming the high polyphenols oil constituents of a blend [50]. Focusing on the Italian oils as part of international blends it should be considered that they mainly consist of Coratina based blends [51]. Interestingly, although specific EVOOs components content may vary according to several agronomic [52] and/or processing [53] factors, Coratina based oils generally represent a relevant part of the overall Italian production [50] and are well known for being rich in both oleic and polyphenols [51,52,54].

### 3.4. Supervised Analysis with Coratina and Non-Italian Oils

**Figure 9:**
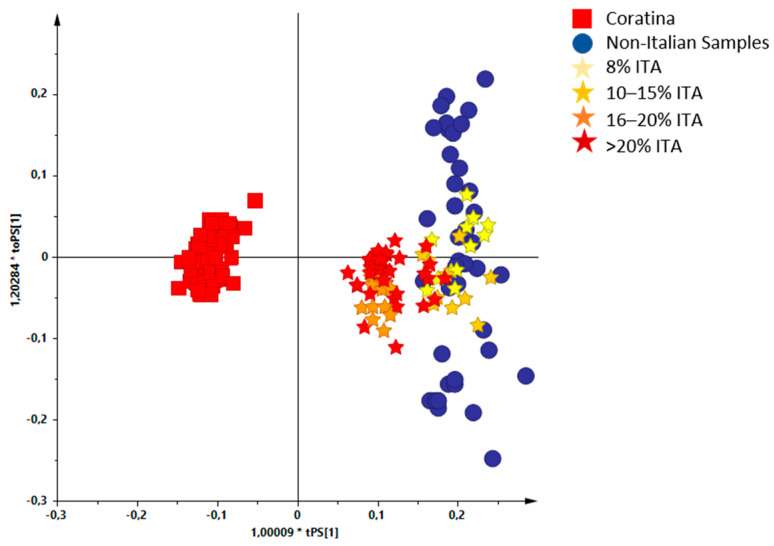
Score plot of the OPLS-DA model for samples of considered organic EU community and non-community oils compared to Coratina and other non-Italian oils; 1 + 1 + 0 components with R^2^X = 0.925, R^2^Y = 0.981 and Q^2^ = 0.974.

As declared by the company supplier, also for the here studied blends the major constituent of Italian oils was a Coratina-based product. Therefore, we predicted the considered organic EVOO blends samples in a two-class OPLS-DA model based on monocultivar Coratina oil samples and non-Italian oils (Spanish, Greek, Portuguese, and Tunisian; Figure 9). The score plot of the OPLS-DA model consists of four components, in which one predictive and three orthogonal, gave R^2^X = 0.925; R^2^Y = 0.981 and Q^2^ = 0.974. According to the values indicated, the supervised model resulted characterized by good predictive power. In the OPLS-DA score plot, the predicted organic EVOO blends (displayed with a color code according to the Italian oil percentage) were clearly positioned in between the monocultivar Coratina oil samples and the non-Italian oils. Interestingly, the resulting position of the international EVOOs in the score plot of the model was clearly collocated along the predictive component, with a proximity to the Coratina samples closely related to the percentage of Italian oil. Nevertheless, the studied international EVOO blends always remain clearly distinct from the Italian Coratina monocultivar oils. These results clearly suggest that the EVOO blends distribution in the score plot may be dictated by the Coratina content in the original Italian oil used for the blend production.

Therefore, it should be also underlined that the methods used here appear to discriminate olive oils based on the contribution of specific varieties rather than direct geographical origins. In the present case, a major contribution of the Coratina variety to the Italian blends was supposed as the reason for the correlation of international blends characteristics with the Italian oil content. Nevertheless, other varieties from different countries, not included in the present study, and characterized by specific marked features, such as a relevant phenolic profile, may give similar correlations when used as international blends constituents.

## 4. Conclusions

In this work, 77 samples of organic EU and outside EU produced EVOO blends, supplied by Certified Origins Italia S.r.l. and originated from the 2017/2018 to 2020/2021 olive harvesting years, were analyzed through ^1^H-NMR spectroscopy associated with multivariate statistical analysis. Both major and minor EVOOs blends components were considered taking advantage of standard and multi-suppressed ^1^H-NMR experiments. These blends include different constituent from specific countries (Italy, Tunisia, Portugal, Spain, and Greece) in different percentages. An unsupervised PCA model obtained with the major components data (^1^H-NMR) showed a clear correlation of the first component (PC1) with the increasing percentage of Italian oil in the blend. The observed progression was not found for the other countries’ components (Tunisia, Portugal, Spain, Greece). When the pairwise joint components percentage was considered, only Italian and Greek components’ joint contribution showed a clear progression along the PC1, possibly due to the very limited Greek oil presence 1–5%. No other clear correlation with PC1 was observed in all the other cases. These results were confirmed by a PLSR analysis where the predicted percentages of Italian oil (according to the model) were reported as a function of the corresponding actual values. The PLSR analysis showed a linear dependence in the range 8–20% ITA and a plateauing effect for higher ITA compositions. Linear progression with PC1 and the plateau were also confirmed with an unsupervised PCA model obtained with the minor components data. The obtained result suggested that oleic acid (standard ^1^H-NMR zg experiment) and polyphenols (fatty acids signals suppression ^1^H-NMR noesy experiment) content, among the major and minor molecular components respectively, gave specific characteristics to the blends. High oleic and polyphenol are specific feature of Coratina EVOO which is the main constituent of Italian oils, as declared by the supplier. Accordingly, the observed trend for the here studied international EVOO blends could be related to the Coratina oil content in the Italian constituents of the blends. Prediction of the international EVOOs in a supervised OPLS-DA model consisting of two-class, monocultivar Coratina oil samples and non-Italian oils (Spanish, Greek, Portuguese, and Tunisian), clearly buttressed this hypothesis. In conclusion, this research underlines that the use of EVOOs with specific marked characteristics such as those of Coratina based Italian oils may be crucial in giving particular features to an international EVOO blend. It should be also taken into account that other cultivars characterized by specific marked features and originating from countries other than Italy may give similar correlations when used as international blends constituents. Moreover, these results confirm that the NMR-bases method, associated with MVA, is a powerful tool to classify commercial oil samples and defining their characteristics also with respect to the blend constituents. Nevertheless, this study is only a preliminary observation referred to 77 commercial samples of organic EU and outside EU EVOO blends. A higher number of commercial samples along with laboratory designed blends with specific composition should be considered for a more detailed investigation of specific constituents’ effects on EVOO blends characteristics.

## Figures and Tables

**Table 1:** Percentages of the oils from different countries making up the organic EU community and non-community EVOO blends. The information of the blends composition was reported on the label of each sample.

Countries	Lower Percentage → Higher Percentage	%
Italy	8 → 10 → 12 → 15 → 17 → 18 → 25 → 27 → 34 → 36 → 37 → 40 → 44 → 50 → 55 → 57	%
Tunisia	17 → 18 → 20 → 22 → 25 → 28 → 30 → 31 → 40 → 48 → 58 → 60	%
Portugal	5 → 19 → 22 → 23 → 24 → 25 → 30 → 32 → 35 → 36 → 40 → 42 → 45	%
Spain	1 → 4 → 10 → 17 → 20 → 22 → 23 → 25	%
Greece	1 → 3 → 5	%

**Table 2:** Coefficient of determination R^2^ for each country within the organic EU community and non-community EVOO blends.

Countries	R^2^
%ITA (8–20%)	0.8160
%ITA (8–>20%)	0.4585
%TUN (17–60%)	0.4397
%SPA (0–25%)	0.2019
%GRE (0–5%)	0.2407
%POR (5–45%)	0.1095

## Data Availability

Data contained within the article are available from the authors.

## Supplementary Materials

Figure S1: Score plot of the PCA model of ^1^H-NMR standard experiment data for samples of studied organic EU community and non-community oil showing the samples colour coding according to the increased percentages of the specific considered oil(s); 3 components model R2X = 0.854, Q2 = 0.773. Figure S2: Partial least squares regression (PLSR) model for considered organic European community and non-community extra virgin olive oil (EVOO) blend samples as a function of the percentage of Italian oil calculated for the specific range 8–20% (a) and full range (b) ITA, and the correlations observed for all the other countries for full range percentages range: Tunisia (c), Portugal (d), Spain (**e**), Greece (f). Figure S3: (a) Table of chemical shifts assignment of the ^1^H-NMR signals of some compounds considered as major and minor molecular components of studied international EVOOs blends. The signal letters agree with those given in Figure S3 (b) and (c). (b) Representative zg ^1^H-NMR spectra of EVOO sample. Main metabolites considered on this work are indicated. (c) Representative noesygpps ^1^H-NMR spectra of EVOO sample. Main metabolites considered on this work are indicated. Table S1: Fatty acids content calculated for all the 77 commercial international EVOO blends.

## Sample Availability

Samples of the compounds “International EVOO blends” are available from the authors.
