# Supplementary material for: 1H-NMR Profiling Shows as Specific Constituents Strongly Affect the International EVOO Blends Characteristics: The Case of the Italian Oil
Source: Molecules. 2021 Apr 13;26(8):2233. doi: 10.3390/molecules26082233 (PMC8069555; doi:10.3390/molecules26082233)
Supplement: Supplementary file 1 [file molecules-26-02233-s001.pdf]

Supplementary

^{1}H-NMR Profiling Shows as Specific Constituents Strongly Affect the International EVOO Blends Characteristics: The Case of the Italian Oil

Francesca Calò [1], Chiara Roberta Girelli [1], Federica Angilè [1], Laura Del Coco [1], Lucia Mazzi [2], Daniele Barbini [2] and Francesco Paolo Fanizzi [1,*]

[1] Department of Biological and Environmental Sciences and Technologies, University of Salento, Prov.le Lecce-Monteroni, 73100 Lecce, Italy; francesca.calo@unisalento.it (F.C.); chiara.girelli@unisalento.it (C.R.G.); federica.angile@unisalento.it (F.A.); laura.delcoco@unisalento.it (L.D.C.)

[2] Certified Origins Italia S.r.l., Località il Madonnino, 58100 Grosseto, Italy; lucia.mazzi@oleificioolma.it (L.M.); daniele.barbini@certifiedorigins.it (D.B.)

* Correspondence: fp.fanizzi@unisalento.it; Tel.: +39-0832-29265

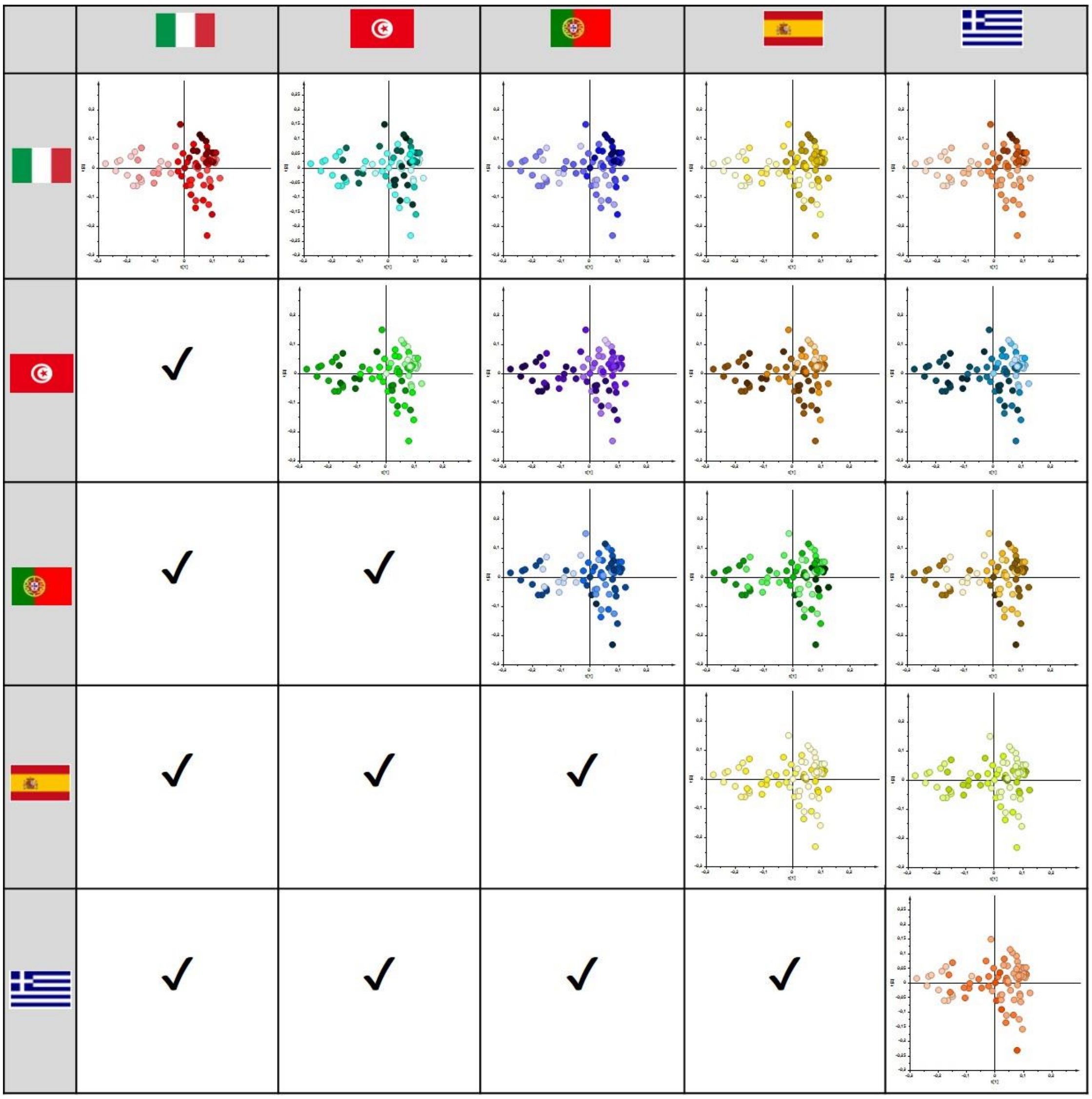

Figure S1: Score plot of the PCA model of ^{1}H-NMR standard experiment data for samples of studied or-ganic EU community and non-community oil showing the samples colour coding according to the increased percentages of the specific considered oil(s); 3 components model R2X = 0.854, Q2 = 0.773.

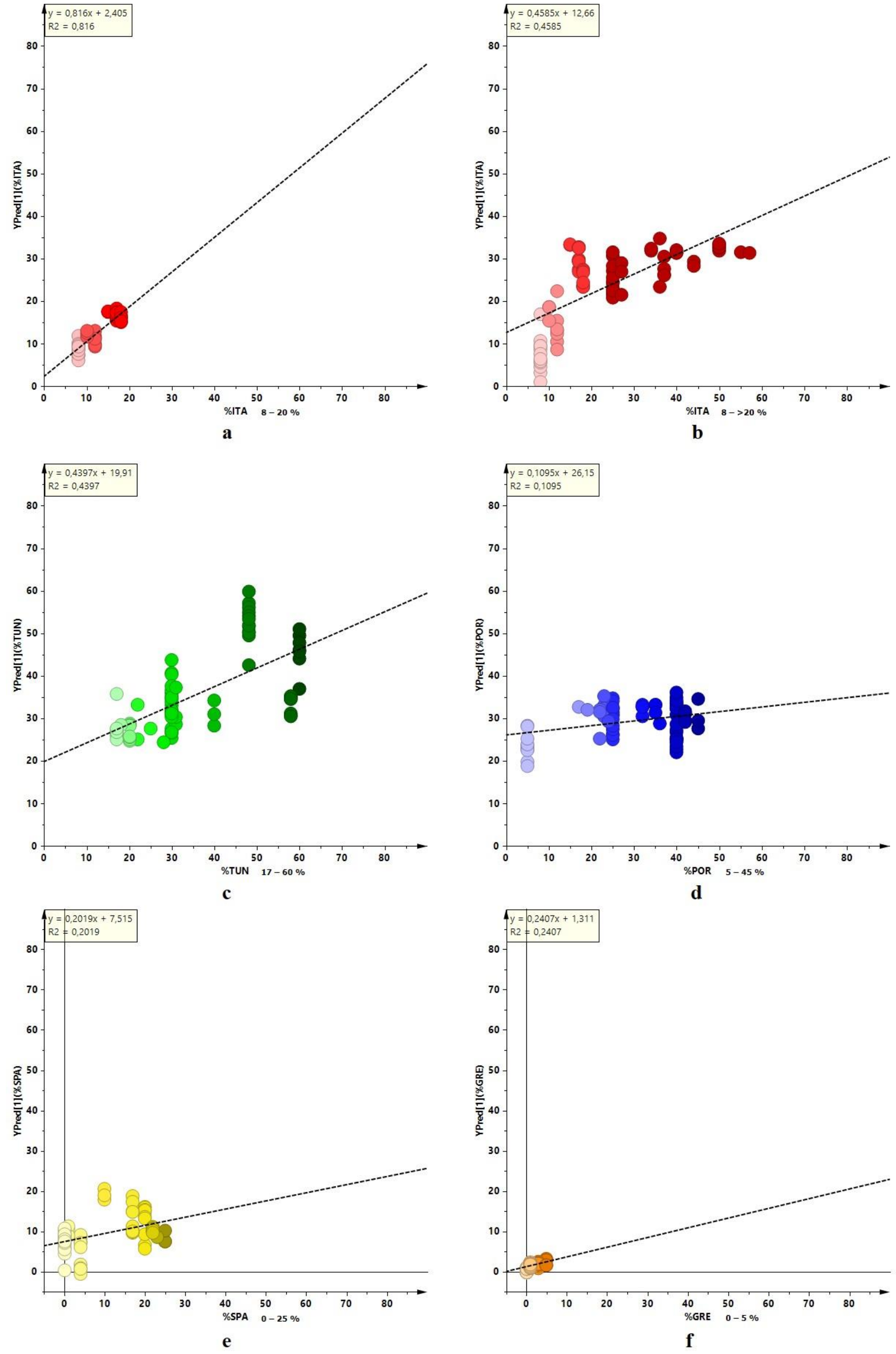

Figure S2: Partial least squares regression (PLSR) model for considered organic European community and non-community extra virgin olive oil (EVOO) blend samples as a function of the percentage of Italian oil calculated for the specific range 8-20% (**a**) and full range (**b**) ITA, and the correlations observed for all the other countries for full range percentages range: Tunisia (**c**), Portugal (**d**), Spain (**e**), Greece (**f**).

a

Signal	Compound	Chemical shift (ppm)	Functional group
**Major component**			
A	Oleic acid	1.03	*-CH_2-*
B	Saturated fatty acids	1.26	*-CH_2-*
C	Oleic acid	2.02	-CH_2-CH=CH-
D	Unsaturated fatty acids	5.34	*-CH=CH-*
**Minor component**			
a	(Z, E) and (E, E) conjugated double bonds associated with hydroperoxiders (OOH)	5.7<br>5.74<br>6.58	*CH=CH-CH=CH-*
b	Tyrosol, Hydroxytyrosol and their derivatives	6.78	*-CH=* (C-7)
c	Oleocanthal (p-HPEA-EDA)	9.22	*-CHO*
d	Elenolic acid	9.62	*-CHO*

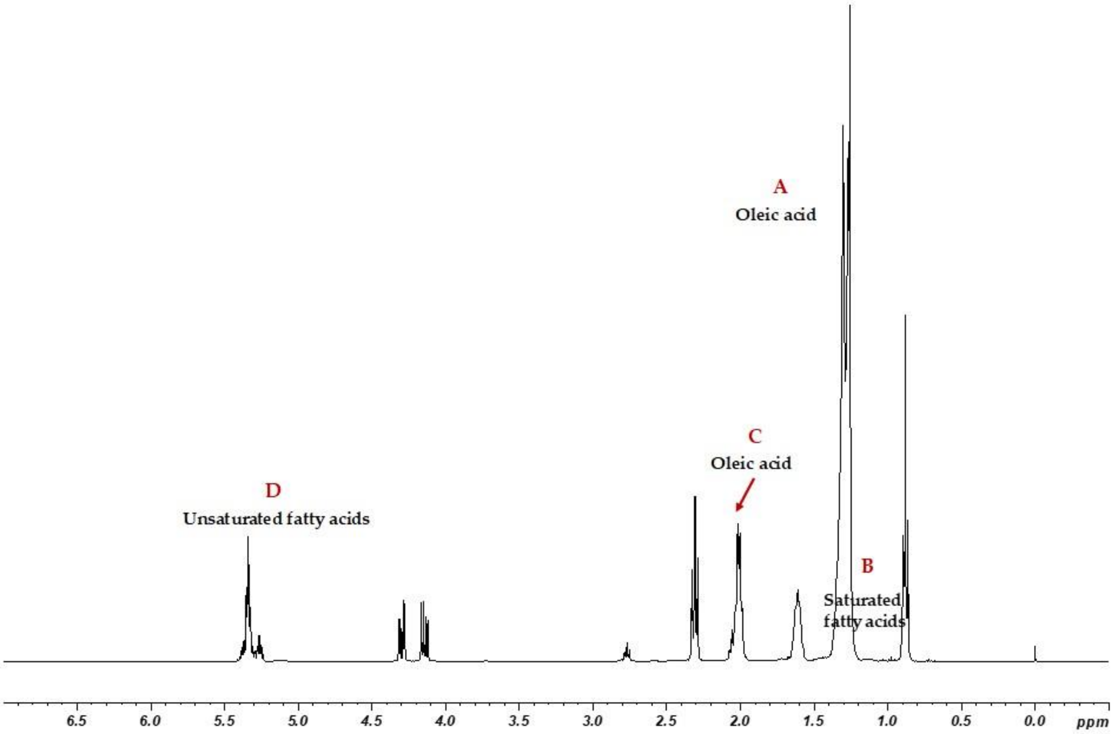

b

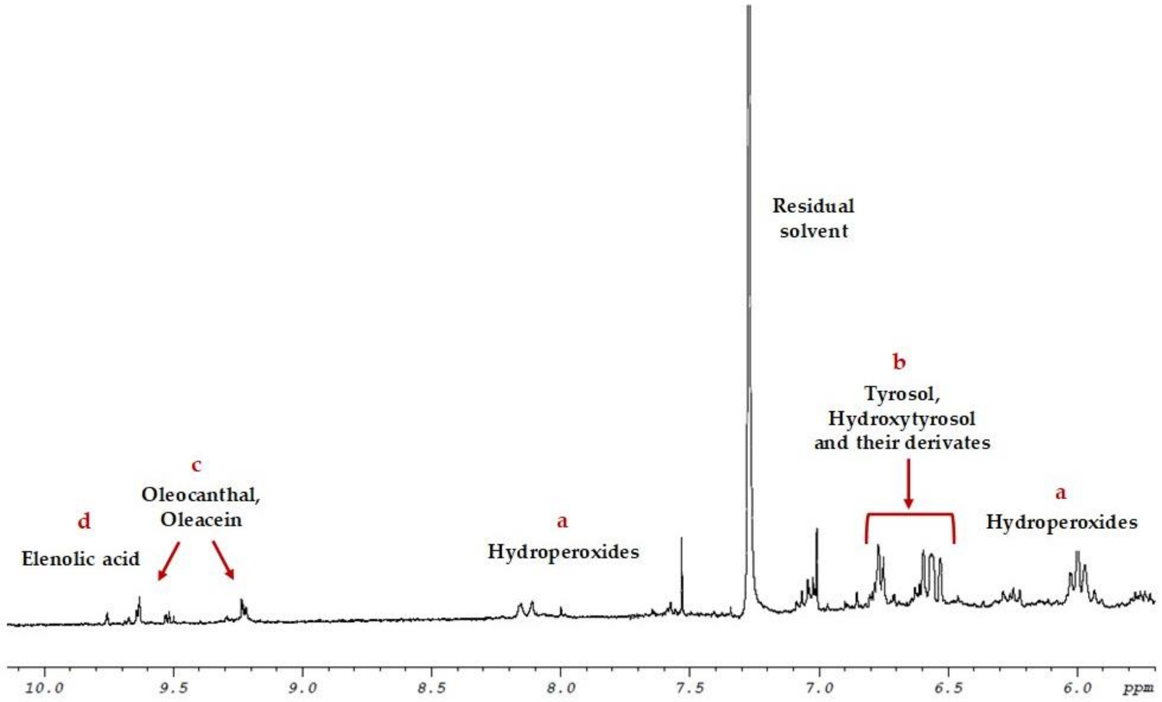

c

Figure S3: (**a**) Table of chemical shifts assignment of the ^{1}H-NMR signals of some compounds considered as major and minor molecular components of studied international EVOOs blends. The signal letters agree with those given in Figure S3 (**b**) and (**c**). (**b**) Representative zg ^{1}H-NMR spectra of EVOO sample. Main metabolites considered on this work are indicated. (**c**) Representative noesygpps ^{1}H-NMR spectra of EVOO sample. Main metabolites considered on this work are indicated.

N°	International EVOO Blends	Harvest Years	Oleic	Linoleic	Linolenic	Saturated
**1**	8%ITA_48%TUN_40%POR_4%SPA	2017/18	66.3	14.9	0.8	19.3
**2**	8%ITA_48%TUN_40%POR_4%SPA	2017/18	66.8	14.5	0.8	19.3
**3**	8%ITA_48%TUN_40%POR_4%SPA	2017/18	69.0	13.7	0.7	18.0
**4**	8%ITA_48%TUN_40%POR_4%SPA	2017/18	64.8	15.9	0.8	20.0
**5**	8%ITA_48%TUN_40%POR_4%SPA	2017/18	67.0	14.5	0.8	19.0
**6**	8%ITA_48%TUN_40%POR_4%SPA	2017/18	68.3	14.1	0.8	18.6
**7**	8%ITA_48%TUN_40%POR_4%SPA	2017/18	68.1	14.1	0.9	18.6
**8**	8%ITA_48%TUN_40%POR_4%SPA	2017/18	71.2	12.4	0.8	17.1
**9**	8%ITA_48%TUN_40%POR_4%SPA	2017/18	68.1	14.1	0.8	18.6
**10**	8%ITA_48%TUN_40%POR_4%SPA	2018/19	67.7	14.7	0.9	18.4
**11**	8%ITA_48%TUN_40%POR_4%SPA	2018/19	66.7	14.9	0.9	19.2
**12**	8%ITA_48%TUN_40%POR_4%SPA	2018/19	67.2	14.3	0.8	19.3
**13**	12%ITA_60%TUN_5%POR_20%SPA_3%GRE	2018/19	69.8	13.3	0.9	17.6
**14**	12%ITA_60%TUN_5%POR_20%SPA_3%GRE	2018/19	70.4	12.9	0.8	17.3
**15**	12%ITA_60%TUN_5%POR_20%SPA_3%GRE	2018/19	69.2	13.7	0.8	17.9
**16**	12%ITA_60%TUN_5%POR_20%SPA_3%GRE	2018/19	67.3	14.4	1.0	19.2
**17**	12%ITA_60%TUN_5%POR_20%SPA_3%GRE	2018/19	68.8	13.6	0.9	18.5
**18**	12%ITA_60%TUN_5%POR_20%SPA_3%GRE	2018/19	73.2	11.1	0.9	16.4
**19**	12%ITA_60%TUN_5%POR_20%SPA_3%GRE	2018/19	68.8	13.9	0.9	18.0
**20**	10%ITA_30%TUN_45%POR_10%SPA_5%GRE	2018/19	69.9	13.2	1.0	17.7
**21**	10%ITA_30%TUN_45%POR_10%SPA_5%GRE	2018/19	71.2	12.4	1.0	17.1
**22**	10%ITA_30%TUN_45%POR_10%SPA_5%GRE	2018/19	71.2	12.4	0.9	17.1
**23**	25%ITA_30%TUN_25%POR_17%SPA_3%GRE	2018/19	71.9	13.5	1.0	15.3
**24**	25%ITA_30%TUN_25%POR_17%SPA_3%GRE	2018/19	71.8	13.7	0.9	15.3

**25**	25%ITA_30%TUN_25%POR_17%SPA_3%GRE	2018/19	71.8	13.6	0.9	15.4
**26**	25%ITA_30%TUN_25%POR_17%SPA_3%GRE	2018/19	71.9	13.5	0.9	15.4
**27**	25%ITA_30%TUN_25%POR_17%SPA_3%GRE	2018/19	73.4	11.9	0.9	15.4
**28**	25%ITA_30%TUN_25%POR_17%SPA_3%GRE	2018/19	72.3	12.4	0.9	16.1
**29**	25%ITA_30%TUN_25%POR_17%SPA_3%GRE	2018/19	73.4	11.8	0.9	15.4
**30**	25%ITA_30%TUN_25%POR_17%SPA_3%GRE	2018/19	74.4	11.0	0.9	15.3
**31**	25%ITA_30%TUN_25%POR_17%SPA_3%GRE	2018/19	74.4	10.9	1.0	15.3
**32**	25%ITA_30%TUN_25%POR_17%SPA_3%GRE	2018/19	72.7	12.1	0.9	16.0
**33**	17%ITA_30%TUN_25%POR_25%SPA_3%GRE	2018/19	74.3	11.1	0.9	15.3
**34**	17%ITA_30%TUN_25%POR_25%SPA_3%GRE	2018/19	74.3	11.1	0.9	15.3
**35**	25%ITA_30%TUN_25%POR_17%SPA_3%GRE	2018/19	75.4	10.4	0.9	14.7
**36**	25%ITA_30%TUN_25%POR_17%SPA_3%GRE	2018/19	75.8	10.5	0.9	14.3
**37**	25%ITA_30%TUN_25%POR_17%SPA_3%GRE	2018/19	74.9	10.5	0.8	15.4
**38**	15%ITA_22%TUN_ 35%POR_25%SPA_3%GRE	2018/19	76.4	9.9	0.9	14.3
**39**	15%ITA_20%TUN_ 40%POR_23%SPA_3%GRE	2018/19	77.0	9.4	0.9	14.2
**40**	17%ITA_20%TUN_40%POR_22%SPA_1%GRE	2019/20	75.0	10.8	0.9	15.0
**41**	17%ITA_20%TUN_40%POR_22%SPA_1%GRE	2019/20	76.5	9.8	0.8	14.4
**42**	17%ITA_20%TUN_40%POR_22%SPA_1%GRE	2019/20	76.5	9.9	0.8	14.4
**43**	17%ITA_20%TUN_40%POR_22%SPA_1%GRE	2019/20	74.9	11.0	0.8	15.0
**44**	17%ITA_20%TUN_40%POR_22%SPA_1%GRE	2019/20	74.9	10.9	0.8	15.0
**45**	17%ITA_20%TUN_40%POR_22%SPA_1%GRE	2019/20	75.0	10.9	0.9	15.0
**46**	17%ITA_20%TUN_40%POR_22%SPA_1%GRE	2019/20	76.6	9.7	0.9	14.4
**47**	17%ITA_20%TUN_40%POR_22%SPA_1%GRE	2019/20	76.5	9.8	0.9	14.4
**48**	27%ITA_31%TUN_40%POR_1%SPA_1%GRE	2019/20	71.5	12.7	0.9	16.5
**49**	27%ITA_31%TUN_40%POR_1%SPA_1%GRE	2019/20	74.2	11.6	0.9	14.8
**50**	27%ITA_31%TUN_40%POR_1%SPA_1%GRE	2019/20	74.1	11.4	0.9	15.4
**51**	18% ITA_58%TUN_23%POR_1%GRE	2019/20	73.7	11.5	0.9	15.4
**52**	18% ITA_58%TUN_23%POR_1%GRE	2019/20	74.0	11.1	0.9	15.7
**53**	18% ITA_58%TUN_23%POR_1%GRE	2019/20	73.8	11.4	0.9	15.4
**54**	18% ITA_58%TUN_23%POR_1%GRE	2019/20	72.2	12.0	0.9	16.3
**55**	18% ITA_58%TUN_23%POR_1%GRE	2019/20	72.2	12.1	0.8	16.3
**56**	18% ITA_58%TUN_23%POR_1%GRE	2019/20	72.2	12.1	0.9	16.3
**57**	18% ITA_58%TUN_23%POR_1%GRE	2019/20	72.2	12.1	0.9	16.3
**58**	37%ITA_40%TUN_22%POR_1%GRE	2020/21	76.2	10.3	0.8	14.4
**59**	37%ITA_40%TUN_22%POR_1%GRE	2020/21	76.0	10.2	0.8	14.7
**60**	37%ITA_40%TUN_22%POR_1%GRE	2020/21	75.0	9.5	0.7	16.5
**61**	50%ITA_30%POR_17%TUN_1%GRE	2020/21	75.3	10.6	0.8	14.8
**62**	50%ITA_17%TUN_32%POR_1%GRE	2020/21	74.1	11.8	0.8	14.9
**63**	50%ITA_17%TUN_32%POR_1%GRE	2020/21	76.2	10.2	0.8	14.4
**64**	50%ITA_17%TUN_32%POR_1%GRE	2020/21	76.2	10.3	0.8	14.5
**65**	50%ITA_17%TUN_32%POR_1%GRE	2020/21	76.1	10.4	0.8	14.5
**66**	34%ITA_30%TUN_35%POR_1%GRE	2020/21	76.5	10.0	0.8	14.3
**67**	34%ITA_30%TUN_35%POR_1%GRE	2020/21	75.7	10.4	0.8	14.6
**68**	36%ITA_28%TUN_35%POR_1%GRE	2020/21	76.3	10.3	0.8	14.3
**69**	44%ITA_30%TUN_25%POR_1%GRE	2020/21	74.3	11.1	0.8	15.4
**70**	44%ITA_30%TUN_25%POR_1%GRE	2020/21	73.7	11.3	0.7	15.7
**71**	55%ITA_25%TUN_19%POR_1%GRE	2020/21	76.1	10.2	0.8	14.7
**72**	57%ITA_18%TUN_24%POR_1%GRE	2020/21	76.5	9.2	0.8	15.2
**73**	40%ITA_17%TUN_42%POR_1%GRE	2020/21	75.6	10.4	0.8	14.9
**74**	40%ITA_17%TUN_42%POR_1%GRE	2020/21	76.1	10.3	0.8	14.6
**75**	40%ITA_17%TUN_42%POR_1%GRE	2020/21	75.7	10.4	0.8	14.8
**76**	37%ITA_22%TUN_36%POR_5%GRE	2020/21	73.7	11.7	0.8	15.7
**77**	36% ITA_17%TUN_42%POR_5%GRE	2020/21	72.4	11.9	0.9	16.5

Table S1: Fatty acids content calculated for all the 77 commercial international EVOO blends.
